# Decoding semiotic minimal genome: a non-genocentric approach

**DOI:** 10.3389/fmicb.2024.1356050

**Published:** 2024-02-27

**Authors:** Carolina Gómez-Márquez, J. Alejandro Morales, Teresa Romero-Gutiérrez, Omar Paredes, Ernesto Borrayo

**Affiliations:** ^1^Biodigital Innovation Lab, Translational Bioengineering Department, Exact Sciences and Engineering University Center, Universidad de Guadalajara, Guadalajara, Mexico; ^2^Technological Innovation Department, Tlajomulco University Center, Universidad de Guadalajara, Guadalajara, Mexico

**Keywords:** minimum genome, non-genocentric, biological information flow, genomic semiotics, language processing tools

## Abstract

The search for the minimum information required for an organism to sustain a cellular system network has rendered both the identification of a fixed number of known genes and those genes whose function remains to be identified. The approaches used in such search generally focus their analysis on coding genomic regions, based on the genome to proteic-product perspective. Such approaches leave other fundamental processes aside, mainly those that include higher-level information management. To cope with this limitation, a non-genocentric approach based on genomic sequence analysis using language processing tools and gene ontology may prove an effective strategy for the identification of those fundamental genomic elements for life autonomy. Additionally, this approach will provide us with an integrative analysis of the information value present in all genomic elements, regardless of their coding status.

## 1 Introduction

Conceptually, *minimal genome* refers to the collection of the genomic information that encodes the fundamental semantic management required to sustain life, whose functional unit, according to general perception, is the cell.

Cell systems are networks of information exchange between genome and metabolism in complex feedback loops. These cell systems support several features that ensure proper cell persistence—e.g., redundancies, convergence, divergence, and other information-handling mechanisms– in different environments, giving the organism robustness and adaptability (Kurasawa et al., 2020). Such information exchange has so far been directly correlated with genome length and complexity (Andrews et al., 2023), which has narrowed the perception of genes as the mechanistic template for molecular –RNA and protein– products and has limited further research to the association between gene quantity and organism complexity.

In their search for the informational network essential core, scientists commenced under the assumption that the minimum number of functions would be equivalent to the minimum number of genes a cell required to sustain life. This genocentric paradigm initially allowed researchers to focus on most of the cellular functions. Nevertheless, this approach has become limited as it considers whole genes as single functional units and disregards mechanisms for gene-expression triggering and other putative functions present in non-translated regions of the genome (Baby et al., 2018; Wang et al., 2018).

Non-translated regions may have a meaningful role in information management. Some have already been described as critical regulatory elements (Andrews et al., 2023) and should, therefore, be included in minimal genome research, which has proved effective in understanding various biological mechanisms, such as the putative bases for biological autonomy, metabolism, adaptation, and evolution. It has also become an important starting point for bioengineering applications –bioproduct synthesis, for example– holding immediate applications in medicine, industry, and environmental sanitation (Lee and Kim, 2020).

In this study, we discuss an alternative perspective to the genocentric paradigm, focusing on DNA as genomic quanta rather than on genes. Therefore, we propose that the minimal genome be the set with the least genomic quanta—regardless of its functional nature—required for an organism to thrive.

## 2 Minimal genome

### 2.1 Minimum biological information flow

The search for the minimal genome became more prominent after JCVI-syn3.0 (Hutchison et al., 2016). Hutchinson, *et al*. achieved reproduction with a significantly smaller genome than any other autonomous organism. As a result, they obtained the minimum number of genes required for cell reproduction, which included known genes and many others whose functions were unknown. The JCVI-syn3.0 model and other efforts performed on natural organisms (Gil and Peret, 2015; Baby et al., 2018) with only essential functions that confer reproductive capability in highly controlled conditions (Baby et al., 2018; Wang et al., 2018) are focused only on the genes involved. Given this scenario, differential gene expression tends to be overlooked, and, consequently, expression regulation elements are usually ignored in minimal genome research. Beyond coding regions, few studies have expanded the minimal genome analysis to non-coding sequences, encompassing life-sustaining essential genomic motifs (Luo et al., 2014; Bartha et al., 2018). These regions include non-translated RNAs as promoters, enhancers, and replication origins (Luo et al., 2014).

Therefore, minimal life-sustaining processes should not only be considered as the genetic expression *per se* but also *when, where*, and *which* genes are to be expressed at any given cellular scenario. It is not likely that genes –as information packages alone– could explain such phenomena. Although survival and reproduction are the main success attributes in terms of minimal genome research, a minimal genome should also consider genomic requirements for cell autonomy, reproduction, adaptation, and evolution. Such processes involve complex interactions among genomic products, sequences, arrangements, and organization, all of which determine *information management*. Furthermore, the challenge of genome minimization resides in furnishing the organism with the “genomic chasis” that allows for its survival in as wide a range of environmental scenarios as possible, enhancing its odds for adaptation and evolution (Moger-Reischer et al., 2023).

### 2.2 The “essentiality” trait

Determining which genes are essential for cellular autonomy and reproduction departed from an intuitive perception of sequential genomic reduction with different analytical approaches [i.e., comparative genomic analyses, gene inactivation studies, and progressive genome reduction (Hutchison et al., 2016; Baby et al., 2018; Dong et al., 2018; Breuer et al., 2019)] that proved less straightforward than initially expected: Some genes that were dispensable when others were present became indispensable when the latter were amiss (Baby et al., 2018; Leeuwen et al., 2020). A further discussion over this has classified gene essentiality into four categories: *no essentiality, low essentiality, high essentiality*, and *complete essentiality*; in accordance with their indispensability for cellular autonomy in different contexts, such as metabolite disposition on media, protein complex redundancy, and alternative metabolic pathways (Rees-Garbutt et al., 2020). Therefore, cellular context highly determines *gene essentiality*, mainly provided by environmental conditions. To adequately categorize genes into such classification, their functionality regarding cellular autonomy should be considered. The analysis of an obligate parasite or a synthetic organism that has specific environmental condition requirements for its autonomy establishes the challenge to determine where to draw the line to consider the organism as *autonomous* (Wang et al., 2018; Breuer et al., 2019). This classification should consider its biological state: whether its single-celled or multi-cellular, and what its environmental and habitat conditions are. Each of these biological states will present different complexity levels in terms of adaptability, *quorum* sensing, and specialization, involving not only genetic expression regulation but also complex information management (Bartha et al., 2018; Rosconi et al., 2022). We consider important to highlight that minimum genome research has been mainly performed on obligate parasites (Gil and Peret, 2015; Baby et al., 2018) and/or in controlled laboratory conditions (Baby et al., 2018; Wang et al., 2018) which do not necessarily represent their natural biological environment and definitively do not involve complex information management.

However, biological information flow analysis should go beyond the binary functionality described by Hutchison et al. (2016) in other biological processes –genome preservation, gene expression, membrane function/structure, and cytosolic metabolism– and be analyzed from a continuous perspective. A well-described example of such continuity is the ribosome competition in regulation control, which implies the importance of a more profound understanding of generally unacquainted information management elements such as riboswitches (Brewer et al., 2021) and other putative non-coding regions.

At a higher level of autonomy, the concept of continuity expands further to address the understanding of adaptation adequately. This approach should include membrane receptors, genetic regulation elements, and related mechanisms that provide energetic optimization. Here, different metabolic pathways are regulated according to a dynamic environment, considering that the cell manages a dynamic information flow within itself (Malerba et al., 2020). We can highlight two evolutionary mechanisms that have been described to expand metabolic capabilities: on the one hand, genetic mutation driven by genetic expansion and selective pressure loss, which provides the opportunity for functionality gain and protein specialization (Noack and Baumgart, 2019; Malerba et al., 2020); on the other hand, genomic reduction, which renders protein multifunctionality (Gil and Peret, 2015). These two different evolutionary processes address information management with different solutions, all of which endorses the concept that information management is an important point of attention in addition to the conventional genomic product analysis.

Following this, the next level would be how a cell interacts with other living organisms present in the environment, either by *Quorum sensing* (Boo et al., 2021), genetic material exchange, or even newly described Borgs (Al-Shayeb et al., 2022). Here, information management acquires a new dimension. Complex interactions now establish an information flow where products from one cell influence the genetic expression of another one, establishing unicellular communities that act as a consortium (Deter and Lu, 2022).

Specialization would be the last level, where cellular compartmentalized organization is now extended to other cells to fully cooperate in a way that each cell plays a determined and committed role as members of a cellular community (Bartha et al., 2018; Kurasawa et al., 2020). At this level, information management involves significantly more complex mechanisms in terms of expression, regulation, and inactivation, all of which modifies cellular behavior (Rosconi et al., 2022), giving rise to multi-cellular organisms.

### 2.3 The non-genocentric search for minimal genome

In Paredes et al. (2018), we explored the way biological phenomena are encoded not only within the genome's coding regions but also in their interactions with non-coding ones. Therefore, we stated the possibility to evidence that the genomic syntax's behavior includes those non-coding genomic regions. In that exploration, the non-coding regulatory genomic regions were disassembled into different size *k-mers*, and the regulatory function's vocabulary diversity among different cellular lines was analyzed. We provided evidence that the lexicon complexity linked to singular vocabularies occurs at a *k-mer* length around 10 nucleotides and that it is at this length where DNA sequences have the degeneracy degree and potential to encode regulatory functions.

In further exploration of genomic lexicon features, we broadened our analysis to all genomic regions, regardless of their coding or non-coding status in bacterial genomes. We screened the lexicons of three bacterial families at a fixed *k-mer* size to evaluate whether genomic organization among bacterial families diverged (Borrayo et al., 2020). We applied topic modeling methods to obtain representative *k-mer* sets for each family and discussed how such divergence in genomic organization could be related to their phenotypical traits.

When COVID strike presented an opportunity to explore not only family lexicon divergence but also mutational phenomena, we replicated the previous approach to hCoV-19 virus sequences (Aguilar-Valdez et al., 2021). We confirmed that *k-mer* sets—or informational compartments—can be linked to particular taxa. We provided evidence that *k-mer* profiles and their organization—*i.e.*, pragmatics—closely reflected phenotypical traits or biological semantics, since a portion of *k-mer* in the virus did not follow expected behavior in a vocabulary arrangement, which in turn corresponded with coronavirus mutational hotspots.

## 3 Discussion

These results led our group to propose a plausible approach to minimum genome identification from a non-genocentric context througth determining the minimum *corpora* –the information-quanta aggregate– to suffice the basic biological needs to be considered autonomous.

Under this perspective, the main challenge lies on identifying essential elements on the complete genome, regardless of their coding or non-coding traditional classification status. This new paradigm allows for both Top-bottom and Bottom-top approximations to a minimum genome (Wang et al., 2018; Rees-Garbutt et al., 2020) can benefit from a shift in focus on how information flow is perceived. Instead of a *gene-to-functional-product* perspective, artificial intelligence tools can be implemented using an *information-based genomic-element analysis* approach to support both types of laboratory procedures.

A non-genocentric approach provides both an integrative analysis of the informational value rendered by the genomic elements involved, and the implementation of information-analysis tools, which have traditionally been applied to other areas, such as natural language processing techniques (Gonzlez-Blas et al., 2019; Kherwa and Bansal, 2020). As genomic information has substantial similarities to natural languages, biological functions can be considered as semantic elements given by *genomic constructions* and not only by *genes*. In turn, these semantic items are driven by an analyzable syntax and vary pragmatically as some genomic constructs can be preferred under determined conditions or be preserved as a remnant of an evolutionary process (Caetano-Anolls, 2021). Therefore, a plausible strategy to address the minimum genome from an information management approach under a non-genocentric paradigm is an adequate use of language processing tools, which have been successfully applied to genomic sequence analysis in different contexts (Gonzlez-Blas et al., 2019; Kherwa and Bansal, 2020).

This strategic use of tools aligns with the broader goals of understanding genomic complexity and optimizing analytical procedures. By implementing these tools, it is possible not only to classify, align, or cluster sequences but also to extract and analyze large amounts of sequence data. The information obtained may in turn establish relationships between mentioned sequences and their related cellular processes, which, along with gene onthology (Carbon et al., 2021; Good et al., 2021), may prove effective in identifying the *corpora* that provide the minimum elements to sustain organism functions at a given autonomy level.

Altogether, genomic elements can be analyzed from a comprehensive point of view, considering their ontological function as their primary attribute and, therefore, disregarding their coding or non-coding status. This consideration could lead to a deeper understanding of the elemental genomic sequences for adequate biological function and regulation. Furthermore, such analysis would render a “minimum genome” with the necessary *corpora* for performing autonomous biological functions in a given environment.

As stated, understanding the genomic minimum requirement for cell autonomy, reproduction, adaptation, and evolution is a key element for applied biotechnology, gene manipulation, protein design, and medical procedures. In addition, this understanding opens new perspectives regarding how life works, how it came to be, and how it copes with information management (Bartha et al., 2018; Hausser et al., 2019; Kurasawa et al., 2020).

**Figure 1:**
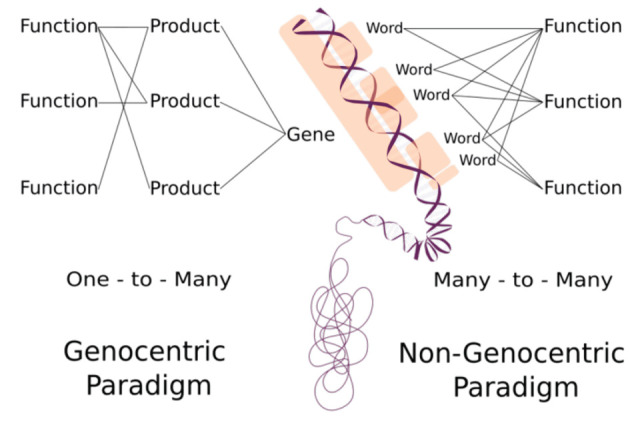
Distinguishing between the genocentric and non-genocentric paradigms involves conceptual differences. The genocentric paradigm adheres to a “one-to-many” relationship, connecting gene to function with a molecular product intermediary. In contrast, the non-genocentric paradigm operates on a “many-to-many” relationship, linking *information quanta* (Words) to function bypassing any product intermediary.

The non-genocentric approach is not intended to substitute the current paradigm in terms of disregarding DNA molecules as the mechanistic template for molecular products. Instead, this approach aims to focus attention on which genomic regions are related to cellular functions. As we shift focus from products to *k-mers*, we are expanding our perception regarding the sequences' role in a particular biological process (Figure 1). The association of functionality between *information quanta* and a specific process potentially expands our understanding and manipulation of genomic information management. This expansion reaches a point where putative functions can be established even if they have not been found in living organisms.

The identification of the particular role of non-coding regions in biological information flow remains as the main challenge to achieve these goals. Potentially, *corpora*-based genomic reduction will establish the core mechanisms in cell systems and provide the scaffold for genetic circuit design implementation, as stated by McBride et al. (2021). Nevertheless, bioengineering has so far focused on genetic edition, neglecting equivalent processes on other types of genomic sequences (Kemp et al., 2020). A plausible solution to address this issue is to further explore how non-coding sequences have influenced the differential in biological complexity among different unicellular kingdoms, which would allow to adequately assign a biological function to generated *corpora* derived from non-coding regions.

Currently, as this approach for a minimal genome develops, we suggest that it be implemented at a single cell autonomy/reproductive level, where all metabolic, regulatory, and information management processes can be supported by experimental data. We look forward to its implementation at higher autonomy levels.

The search for a minimum genome using our approach may render more than one solution, which is congruent with the different paths life has taken to adapt to specific conditions. In addition, it can provide significant insights into the information management basis, which in turn will prove useful in understanding biological functions, evolution, and subsequent applications in medical, environmental, and industrial biotechnologies. It is highly probable that guided *corpora* assembly will become a common bioengineering practice in the near future, either by means of cellular or cell-free system implementation, and through the design of infromation-flow solutions that have not necessarily been obesrved in natural systems.

## Data availability statement

The original contributions presented in the study are included in the article/supplementary material, further inquiries can be directed to the corresponding author.

## Author contributions

CG-M: Conceptualization, Writing—original draft, Writing—review & editing. JAM: Conceptualization, Writing—review & editing. T-RG: Conceptualization, Writing—review & editing. OP: Conceptualization, Writing—review & editing. EB: Conceptualization, Funding acquisition, Supervision, Writing—review & editing.
